# Development of a one-pot RPA-cas12a/13a assay for simultaneous detection of HPV16 and HPV18

**DOI:** 10.3389/fbioe.2025.1608301

**Published:** 2025-07-17

**Authors:** Qiujie Yu, Hongzhi Luo, Xiaoxue Huang, Xiaohua An, Yan Wang, Huasu Chen, Jianhuang Rong, Yafei Zhang, Qianhao Huang, Yudi Rao, He Zha

**Affiliations:** ^1^ Department of Laboratory Medicine, The Third Affiliated Hospital of Zunyi Medical University (The First People’s Hospital of Zunyi), Zunyi, China; ^2^ Scientific Research Center, The Third Affiliated Hospital of Zunyi Medical University (the First People’s Hospital of Zunyi), Zunyi, China; ^3^ Department of Experimental Medicine, Jintang First People’s Hospital·West China Hospital Sichuan University Jintang Hospital, Chengdu, China; ^4^ Department of Oral Maxillofacial Surgery, School and Hospital of Stomatology, Zunyi Medical University, Zunyi, China

**Keywords:** HPV, one-pot reaction, CRISPR-Cas12a/13a, recombinase polymerase amplification, multiplex detection

## Abstract

The incidence of human papillomavirus (HPV)-related cervical cancers has been on the rise, and the affected population is increasingly younger. Early-stage prevention and screening initiatives have emphasized the critical necessity for reliable and rapid HPV detection technique. In this study, we devised a fluorescence-based assay that integrated one-pot Cas12a/13a with recombinase polymerase amplification (RPA) for the detection of HPV16 and HPV18. We exploited the cleavage activities of the Cas12a and Cas13a enzymes to specifically target the L1 gene of HPV16 and 18, respectively. The diagnostic efficacy of the CRISPR-Cas12a/13a system was assessed in identifying HPV by analyzing clinical samples and comparing it with the PCR method. The one-pot RPA-Cas12a/13a-based fluorescence assays exhibited a sensitivity of 10 copies/µL, and required only 40 min for completion. Compared with PCR method, the overall sensitivity and specificity of this assay were 97.69% and 100%, respectively, with a kappa value of 0.967. This study presents a novel approach for cervical cancer screening and HPV infection surveillance, which may hold potential for the early diagnosis and prevention of HPV-related cervical malignancies.

## 1 Introduction

Cervical cancer is the fourth most prevalent and deadly malignancy among women worldwide (Arbyn et al., 2020), posing a significant threat to women’s health. Cervical cancer is strongly linked to the development of human papillomaviruses (HPV) infection (Liu et al., 2022). HPV16 and HPV18 are the most common high risk HPV (HR-HPV) types, accounting for approximately 70% of all cervical cancer cases (Mazarico and Gonzalez-Bosquet, 2012; Mirabello et al., 2017; Sipp et al., 2018). Therefore, testing for HR-HPV can help identify precancerous cellular changes at an early stage, allowing for timely interventions to prevent cancer progression.

To date, various methods for detecting HPV have been developed, with fluorescent PCR being the most commonly utilized technique in clinical practice. Multiplexed fluorescent PCR demonstrates good sensitivity and specificity. However, it necessitates specialized laboratory equipment and typically requires 2–3 h to yield results (Li et al., 2020). Thus, the development of simple, rapid, accurate, and low-cost diagnostic methods for detecting HPV16/18 is crucial for facilitating the early detection of precancerous cells, enabling timely treatment and prevention of cancer progression.

In recent years, several nuclease-based viral assays have been developed (Xun et al., 2021; Liu et al., 2025). Among them, diagnostic techniques based on the CRISPR/Cas system have gained widespread attention (Manghwar et al., 2019). The most widely studied CRISPR proteins are Cas9, Cas12a, and Cas13a. Both Cas12a and Cas13a can specifically cleave target sequences and non-specifically cleave single-stranded nucleic acids [8]. Cas12a and Cas13a non-specifically cleaves single-stranded DNA (ssDNA) and single-stranded RNA (ssRNA), respectively (Kellner et al., 2019; Hu et al., 2022).

Currently, the CRISPR-Cas12a/13a system has been utilized to detect a wide range of pathogens, including Dengue virus (DENV), HPV, and Methicillin-Resistant *Staphylococcus aureus* (Zheng et al., 2022; Liu et al., 2023; Tian et al., 2024). However, these methods generally require two separate steps for the CRISPR dual system and amplification reactions, typically performed in distinct reaction tubes, which raises concerns about the potential for aerosol contamination and increases the complexity of the procedure. Tan et al. employed the CRISPR-Cas12a/13a system to detect tumor markers (Tan et al., 2024). But its sensitivity is low. RPA, a widely used isothermal amplification technique, operates at 37°C–42°C, requires smaller sample volumes, and can amplify DNA targets as low as 1–10 copies within 20 min (Tan et al., 2022). The combination of RPA and CRISPR systems facilitates sensitive, accurate, and rapid detection.

In this study, we have established a one-pot RPA-Cas12a/13a assay to detect HPV 16/18 simultaneously. The cleavage system utilizing Cas12a for detecting the HPV16 and Cas13a for detecting the HPV18 (Primers incorporating the T7 promoter sequence were added). To avoid aerosol contamination and simplify the process, we combined multiplex RPA amplification with a CRISPR dual-channel detection system. We detected the HPV16/18 L1 genes simultaneously. The development of this method offers novel technical support for the early screening of cervical cancer and the detection of HPV infection.

## 2 Materials and methods

### 2.1 Materials and reagents

LbaCas12a, 10×NEBuffer 2.1, T7 RNA polymerase, and rNTP mix were obtained from New England Biolabs. LwaCas13a protein was obtained from Bio-Lifesci Co., Ltd. RNase inhibitors were sourced from Vazyme Biotech Co., Ltd. RPA amplification was performed using the DNA Thermostat Rapid Amplification Kit (Basic), bought from Amp-Future Biotech Co., Ltd. All primers, a single-stranded DNA fluorescent probe, and HPV16 L1 gene and HPV18 L1 gene plasmids were bought from Shanghai Sangon Biotech Co., Ltd. A single-stranded RNA fluorescent probe and all crRNAs were obtained from Accurate Biotechnology (Hunan) Co., Ltd.

### 2.2 Primers and crRNA design and synthesis

The sequences of HPV16/18 L1 gene were compared in Gene bank database and NCBI BLAST was used to find highly conserved sequences of HPV16/18 L1 gene. HPV16 L1 gene sequence (GenBank: MG850145.1), HPV18 L1 gene sequence (GenBank: LC509006.1) were downloaded from the NCBI database (https://www.ncbi.nlm.nih.gov/nuccore/). According to TwistAmp’s RPA primer design guidelines, the corresponding RPA primers were designed in the conserved regions of the target genes. The IDT Oligo Analyzer website (https://sg.idtdna.com/site/home/home/sessiontimeout) was used to verify that the primers conformed to each design principle and to screen for the best primer pairs. The species specificity of the sequences was validated through the use of Primer-BLAST on the NCBI website (https://www.ncbi.nlm.nih.gov/tools/primer-blast), ensuring that the sequences were specific to the target species. The crRNA for Cas12a and Cas13a was designed based on the cleavage characteristics of the respective Cas proteins. The full list of oligonucleotides used in this study is provided in Table 1.

**TABLE 1:** The oligonucleotides used for RPA and crRNA in CRISPR-based assay for HPV 16/18 detection.

Oligonucleotides	Sequence (from 5′ to 3′)
Cas13a-HPV18-crRNA	GAU​UUA​GAC​UAC​CCC​AAA​AAC​GAA​GGG​GAC​UAA​AAC​UCA​GAG​GUA​ACA​AUA​GAG​CCA​CUU​GGA​GUC​CCC​AAA​UGA​GUA​UUU​UUC​UUU​AAC​UG
Cas12a-HPV16-crRNA	UAA​UUU​CUA​CUA​AGU​GUA​GAU​GCC​AGU​UCA​AAU​UAU​UUU​CC
RNA reporter	FAM-UUUUU-BHQ1
DNA reporter	ROX-TTATT-BHQ2
T7 promoter	GAA​ATT​AAT​ACG​ACT​CAC​TAT​AGG​G
HPV16-RPA-F1	AGA​CGA​TTT​ATA​CAT​TAA​AGG​CTC​TGG​GTC
HPV16-RPA-R1	TGT​AGT​TTC​TGA​AGT​AGA​TAT​GGC​AGC​AC
HPV16-RPA-F2	ACG​ATT​TAT​ACA​TTA​AAG​GCT​CTG​GGT​CT
HPV16-RPA-R2	GTA​GTT​TCT​GAA​GTA​GAT​ATG​GCA​GCA​CAT
HPV16-RPA-F3	CGA​TTT​ATA​CAT​TAA​AGG​CTC​TGG​GTC​TAC
HPV16-RPA-R3	GTT​TCT​GAA​GTA​GAT​ATG​GCA​GCA​CAT​AAT
HPV18-RPA-F1	GAA​ATT​AAT​ACG​ACT​CAC​TAT​AGG​GTA​TGG​GTG​ACA​CTG​TGC​CTC​AAT​CCT​TAT​A
HPV18-RPA-R1	CAC​CAA​TAA​CCT​CAT​ATC​CAA​CAT​CAC​AGT
HPV18-RPA-F2	GAA​ATT​AAT​ACG​ACT​CAC​TAT​AGG​GCT​ATG​GGT​GAC​ACT​GTG​CCT​CAA​TCC​TTA​T
HPV18-RPA-R2	CAC​CAA​TAA​CCT​CAT​ATC​CAA​CAT​CAC​AGT
HPV18-RPA-F3	GAA​ATT​AAT​ACG​ACT​CAC​TAT​AGG​GAC​TAT​GGG​TGA​CAC​TGT​GCC​TCA​ATC​CTT​A
HPV18-RPA-R3	AAC​ACC​AAT​AAC​CTC​ATA​TCC​AAC​ATC​ACA

### 2.3 RPA amplification system

The RPA reaction was performed using the Amp-Future reagent kit, with the following modifications according to the instructions. Add 29.4 µL of A buffer, 14.1 µL of nuclease-free water, and 2 μL of primers (10 µM) to the lyophilized powder tube. Mix thoroughly to dissolve the powder, cap the tube, and briefly centrifuge to collect the reagents at the bottom. After mixing, the RPA mixture and 2 μL of DNA and B buffer (MgCl_2_) were added to the bottom of reaction tube. After centrifugation and mixing, add an appropriate amount of paraffin oil to cap the tube and prevent aerosol formation. The reaction was immediately incubated at 37°C for 20 min. At the end of the reaction, the amplification products were purified using a Tris-saturated phenol/chloroform/isoamyl alcohol (25: 24: 1) extraction, and the results were visualized by electrophoresis on a 2% agarose gel. Set the voltage to 120 V and run for 30 min. After electrophoresis, place the gel in a UV gel imaging system to observe the bands. The multiplex RPA reaction is carried out following the same procedure as previously described, with the sole difference being the inclusion of two primer pairs. Each primer was used at a concentration of 10 μM, and 2 µL of each primer was added to the reaction.

### 2.4 CRISPR dual channel system response

In the one-pot platform, the RPA mix was added to the bottom of the PCR tube (as described above), the reaction solution was incubated at 37°C for 20 min. At the end of the RPA reaction, the CRISPR reagent, preloaded on the cap of the tube, was centrifuged and mixed with the RPA amplification reagent. CRISPR reaction system: LwaCas13a (50 nM), Cas13a-crRNA (50 nM), ROX-ssRNA-BHQ2 probe (250 nM), RNase inhibitor, rNTP mix (1 mM), T7 RNA polymerase (1U/µL), LwaCas12a (266 nM), Cas12a-crRNA (266 nM), FAM-ssDNA-BHQ1 probe (800 nM) in a total volume of 15 uL. The solutions were mixed and centrifuged before being promptly transferred to the PCR instrument. Simultaneous detection of fluorescent signals emitted from the FAM and ROX channels.

### 2.5 Sensitivity and specificity analysis

The HPV16/18 plasmids were diluted in a gradient from 10^5^ to 10^0^ copies/μL for sensitivity assessment. The viral DNA of HPV33, 52, 39, 51, 58 and the RNA of HCV were used to assess cross-reactivity.

### 2.6 Clinical sample collection and DNA extraction

The specimens of human vaginal secretions used in this study were provided by the Third Affiliated Hospital of Zunyi Medical University (the First People’s Hospital of Zunyi), with the sampling period ranging from May 2024 to December 2024. The age range of the subjects was 18–84 years old. And the inclusion criteria were as follows: the subjects were not pregnant or menstruating, had not engaged in sexual intercourse within 24 h, and had not undergone vaginal irrigation or taken any vaginal medications within 72 h. Clinical samples were evaluated using the widely used PCR probe method as the gold standard. The samples collected in this study were approved by the Ethical Review Board of the Third Affiliated Hospital of Zunyi Medical University. We used an automated nucleic acid extractor to extract nucleic acids. The experimental procedures are as follows: First, the samples were shaken thoroughly to achieve homogeneous mixing, and then the plastic sealing film of the nucleic acid extraction kit was removed. Subsequently, 20 µL of proteinase K and 300 µL of the samples were added. Next, nucleic acid extraction was carried out on the automatic nucleic acid extractor using the set program. After extraction, the obtained nucleic acids were utilized for PCR amplification.

### 2.7 The qPCR reaction system and parameters

HPV16/18 was detected using the Human Papillomavirus Nucleic Acid Typing Kit (PCR-Fluorescent Probe Method) from Sansure Biotech Inc. The PCR system was 38 uL of PCR reaction solution, 2 uL of enzyme mix, 10 uL of nucleic acid, and the total volume of the reaction was 50 uL. The detection was carried out using the SLAN 96P Real-Time PCR system (Shanghai Hongshi Medical Treatment Technology Co., Ltd., Shanghai, China). And the procedure was as follows: the first step was UDG enzyme reaction at 50°C for 2 min; the second step was Taq enzyme activation at 94°C for 5 min; the third step was amplification reaction for 45 cycles (94°C for 15 s, 57°C for 30 s); the fourth step was cooling at 25°C for 10 s.

### 2.8 Statistical analysis

In this study, we used “Normalized PL Intensity” as the expression of fluorescence signal data. For two groups of data with normal distribution, we used the t-test for statistical analysis. For comparison between multiple groups of data, we used one-way ANOVA. All experiments were conducted in triplicate and analyzed using GraphPad Prism version 10.1.2 (GraphPad Software, Inc.). And we used Graphpad (https://www.graphpad.com/quickcalcs/kappa1/) to calculate the kappa value of one-pot RPA-Cas12a/13a to assess its credibility. Kappa value <0: No agreement, Kappa value between 0.00 and 0.20: Slight agreement, Kappa value between 0.21 and 0.40: Fair agreement, Kappa value between 0.41 and 0.60: Moderate agreement, Kappa value between 0.61 and 0.80: Substantial agreement, Kappa value between 0.81 and 1.00: Almost perfect agreement.

## 3 Results

### 3.1 Simultaneous detection of HPV genotype 16/18 by one-pot assay principle

**FIGURE 1:**
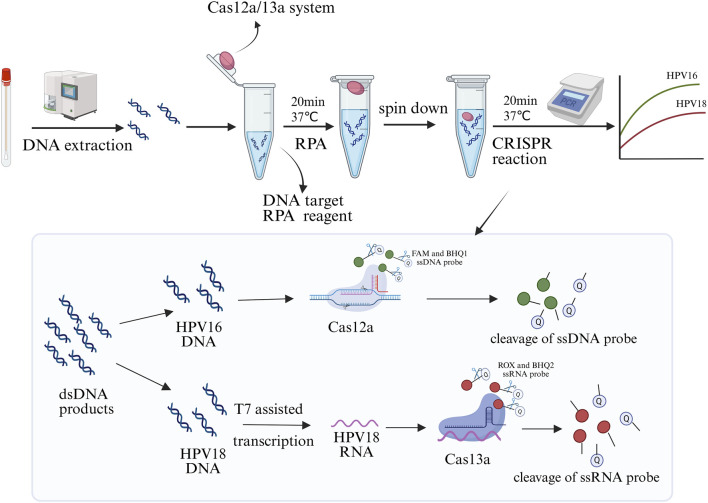
Schematic diagram of the one-pot RPA-Cas12a/13a assay for the simultaneous detection of HPV16 and HPV18. The CRISPR system was placed at the cap of the tube, while the RPA components were added to the bottom for amplification at 37°C for 20 min. After brief centrifugation, the CRISPR system was transiently dissociated to the bottom of the tube to react with the amplification products for 20 min, enabling readout of real-time fluorescence signals in optical instruments. Created in BioRender. Qiujie, Y. (2025) https://BioRender.com/m60g599.

The principle of the one-pot RPA-Cas12a/13a assay for simultaneous detection of HPV16 and HPV18 is illustrated in Figure 1. Multiplex RPA amplifies the target sequences of HPV16 and HPV18, and the Cas12a/crRNA1 and Cas13a/crRNA2 complexes specifically recognize the L1 genes of HPV16 and HPV18, respectively, triggering the trans-cleavage activity of the CRISPR-Cas system. Once the Cas proteins are activated, CRISPR-Cas13a exhibits a strong response to the ssRNA reporter (ROX-UUUUU-BHQ1) by non-specifically cleaving it. Meanwhile, activated CRISPR-Cas12a cleaves the ssDNA reporter (FAM-TTATT-BHQ2). This system enables simultaneous detection of two HPV types in a single reaction, with separate fluorescent signals for HPV16 and HPV18, ensuring specificity and efficiency.

### 3.2 Optimizing the RPA system

In order to get better amplification efficiency, multiple primer pairs were designed for each gene following the principles of RPA primer design. After RPA amplification, the amplification products were subjected to agarose gel electrophoresis to verify the amplification efficiency. As shown in Supplementary Figure S1, All primer pairs for HPV18, the bands in lane 4 exhibited the highest intensity (Supplementary Figure S1A). Similarly, all three primer pairs successfully amplified the HPV16 L1 gene. The HPV16 F3/R3 primer pair (lane 3) demonstrated the highest amplification efficiency (Supplementary Figure S1B). Consequently, HPV18 F2/R1 and HPV16 F3/R3 were selected for subsequent experiments.

**FIGURE 2:**
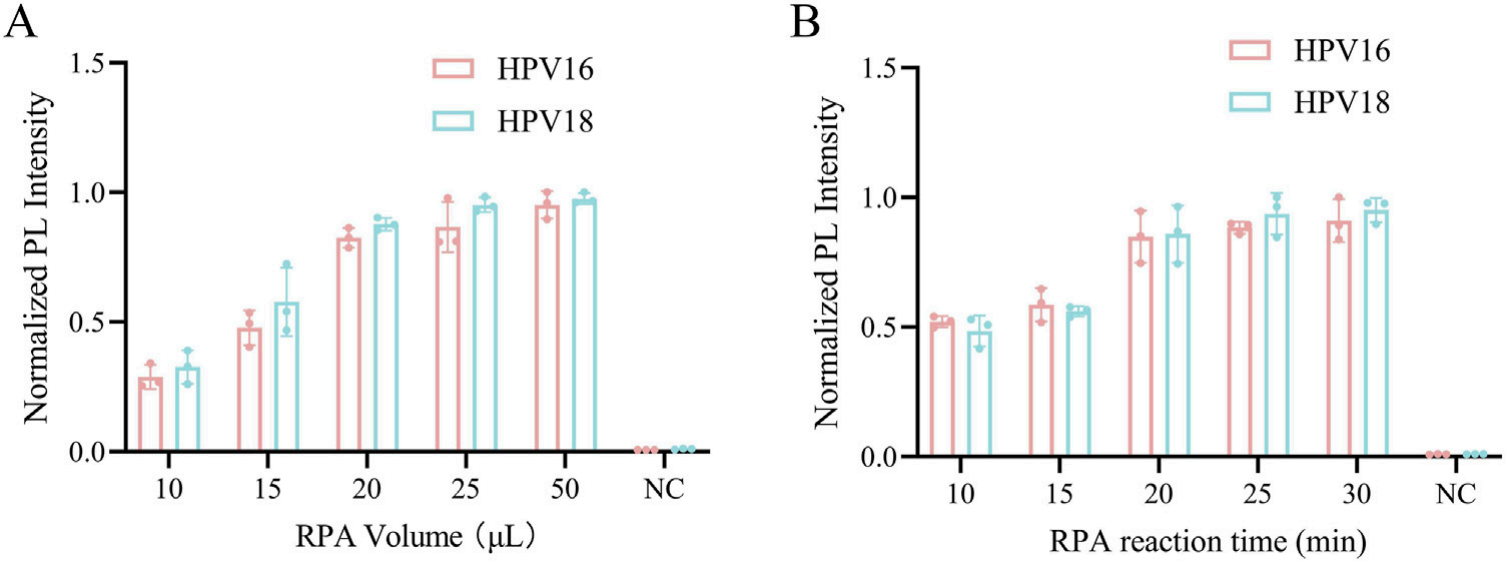
Optimization of the RPA reaction system. **(A)** The RPA reaction volume was optimized from 10, 15, 20, 25, and 50 μL. **(B)** The RPA reaction time was optimized from 10, 15, 20, 25 and 30 min. NC indicates nuclease-free water.

In the optimization of the one-pot RPA-Cas12a/13a assay, we investigated the impact of different RPA volumes and reaction times on fluorescence intensity. Our results showed that RPA volumes of 20 μL, 25 μL, and 50 μL generated stronger fluorescence, with no statistically significant difference between the three volumes (Figure 2A). The 20 μL volume was chosen to reduce reagent consumption while maintaining satisfactory assay performance. Additionally, the reaction time of RPA is also an important influencing factor of amplification, and we set up five subgroups of 10, 15, 20, 25, and 30 min to detect fluorescence signals according to the conditions of RPA amplification. Fluorescence intensity gradually increased as the reaction progressed and stabilized around 20 min (Figure 2B). Thus, 20 μL of RPA volume and 20 min was determined to be the optimal reaction condition.

### 3.3 Platform feasibility assessment

**FIGURE 3:**
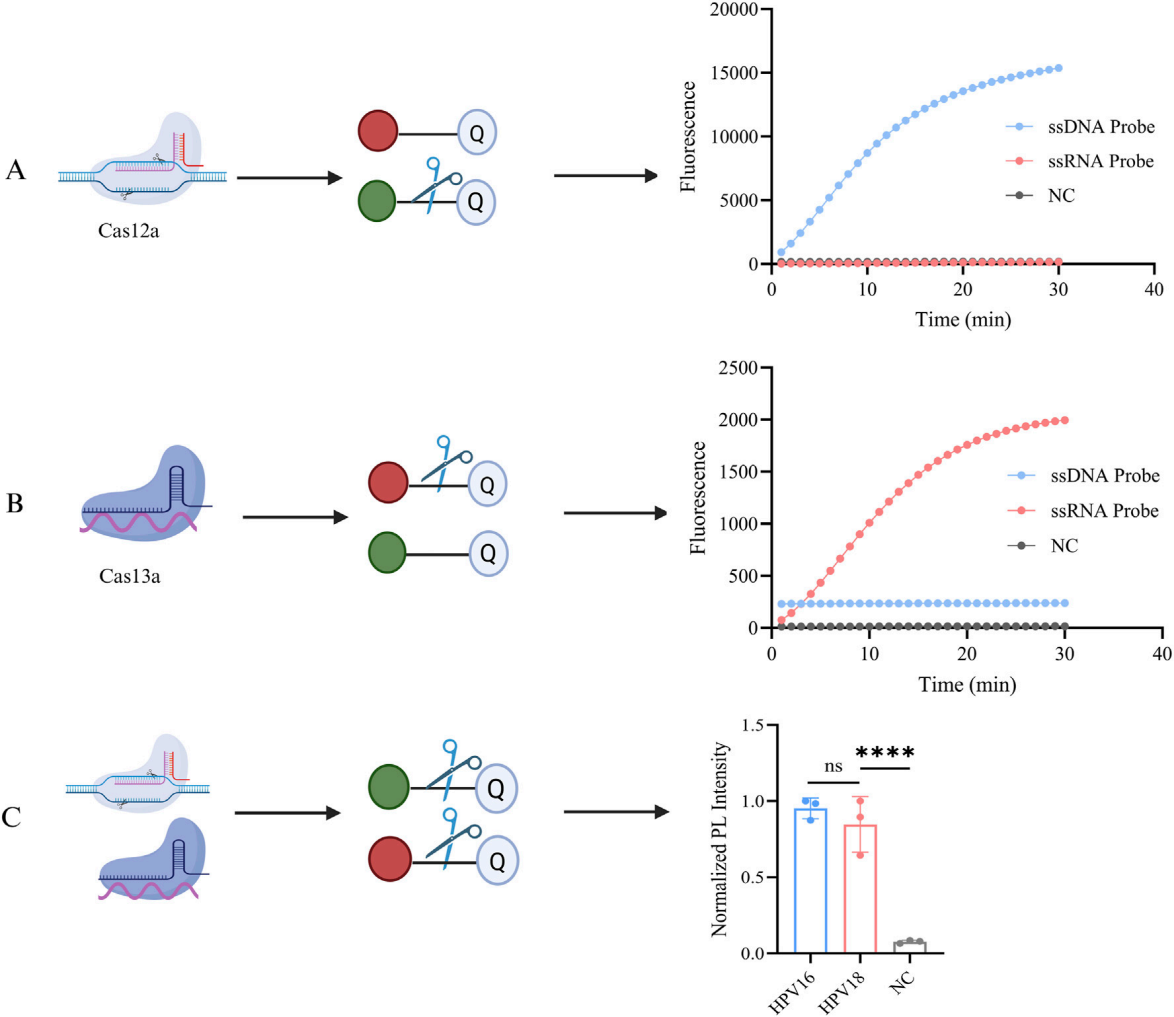
Orthogonal experiments with the CRISPR system. **(A)** Both ssDNA reporter (FAM-BHQ) and ssRNA reporter (ROX-BHQ) were added to the detection systems of Cas12a, and changes in the fluorescence intensity of both types were observed. The activated Cas12a cleaved only the ssDNA reporter, generating a FAM fluorescent signal. **(B)** Both ssDNA reporter (FAM-BHQ) and ssRNA reporter (ROX-BHQ) were added to the detection systems of Cas13a, and changes in the fluorescence intensity of both types were observed. The activated Cas13a cleaved only the ssRNA reporter, generating a ROX fluorescent signal. **(C)** The schematic diagram of the dual-gene detection system for HPV16/18 with LbaCas12a and LwaCas13a. NC indicates nuclease-free water (****P < 0.0001). Created in BioRender. Qiujie, Y. (2025) https://BioRender.com/m60g599.

We constructed a one-pot Cas12a/13a dual-channel detection system for the simultaneous detection of HPV16 and HPV18 L1 genes. To validate the previously demonstrated cross-reactivity of the CRISPR-Cas12a/13a system, orthogonal experiments were performed. The ssRNA reporter (ROX-BHQ) was added to the Cas12a detection system, while the ssDNA reporter (FAM-BHQ) was added to the Cas13a cutting system. A fluorescence PCR instrument was used to simultaneously monitor fluorescence signals from both the FAM and ROX channels. As shown in Figures 3A,B, the dual CRISPR system exhibited excellent orthogonal detection ability without cross-interference, with Cas12a exclusively cleaving DNA and Cas13a exclusively cleaving RNA. When both probes and Cas systems were present, simultaneous detection of both genes in a single tube was achievable (Figure 3C).

### 3.4 Optimized parameters for dual-channel detection systems

**FIGURE 4:**
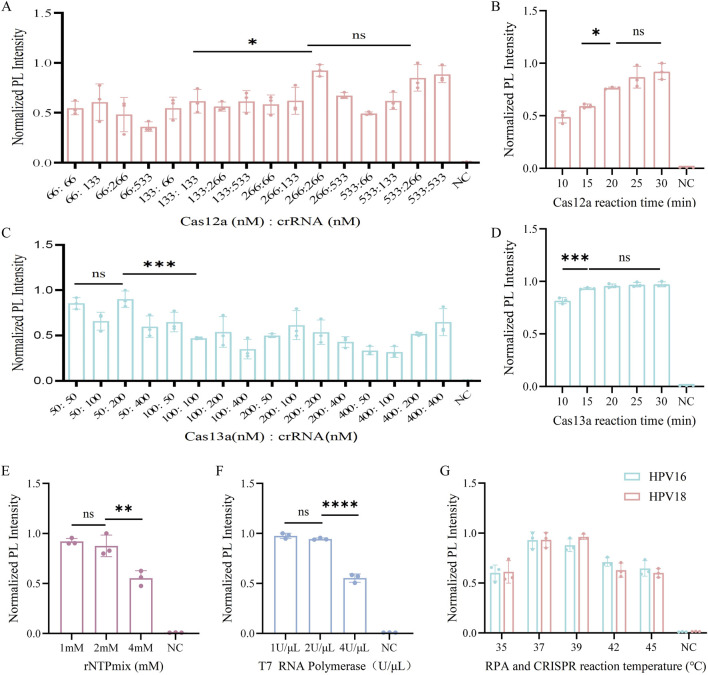
Optimization of the CRISPR system. **(A)** Optimization of Cas12a to crRNA concentration ratio for the Cas12a system in HPV16 L1 genetic testing. **(B)** Optimization of the reaction time of CRISPR/Cas12a from 10, 15, 20, 25 and 30 min. **(C)** Optimization of Cas13a to crRNA concentration ratio for the Cas13a system in HPV18 L1 genetic testing. **(D)** Optimization of the reaction time of CRISPR/Cas13a from 10, 15, 20, 25 and 30 min. **(E)** Optimization of rNTPMix solution concentration in the Cas13a cleavage system. **(F)** Optimization of T7 RNA polymerase concentration in the Cas13a cleavage system. **(G)** Optimization of RPA and CRISPR reaction temperatures from 35, 37, 39, 42, 45°C. NC indicates nuclease-free water (ns > 0.05, *P < 0.05, *P < 0.01, ***P < 0.001, ****P < 0.0001).

First, we optimized the cutting system of Cas12a. To determine the optimal Cas12a-to-crRNA concentration ratio, reaction conditions with ratios ranging from 66, 133, 266, to 533 nM were evaluated. When the ratio was 1:1 (266 nM: 266 nM), strong cleavage efficiency was observed and increasing the concentration of Cas12a or crRNA Cas12a cleavage efficiency did not increase (Figure 4A). This concentration was chosen for the subsequent dual-channel assay. Subsequently, we investigated the optimal reaction time for Cas12a, and similarly set five reaction times and observed in the results that the fluorescence intensity was increasing with time, and fluorescence intensity was observed to stabilize after 20 min of cleavage, establishing 20 min as the optimal reaction time (Figure 4B). And the increase in fluorescence intensity was not statistically significant as the reaction time increased, which indicated that the reaction reached equilibrium.

We then optimized the key parameters of the Cas13a cleavage system. When the Cas13a to crRNA concentration ratio was 1:1 (50 nM: 50 nM), strong cleavage efficiency was achieved (Figure 4C). As shown in Figure 4D, the fluorescence intensity began to increase at 10 min and stabilized after 15 min. To ensure optimal cleavage efficacy for both Cas12a and Cas13a in the one-pot system, 20 min reaction time was chosen for the CRISPR system. We Optimized of the rNTP mix concentration revealed no statistically significant difference in fluorescence produced by the ROX channel between 1 mM and 2 mM rNTP mix concentrations. Therefore, we selected a concentration of 1 mM for subsequent experiments (Figure 4E). Additionally, we optimized the concentration of T7 RNA polymerase, another key parameter in the Cas13a system. There was no statistically significant difference in fluorescence produced by the ROX channel at 1 U/μL and 2 U/μL concentrations (Figure 4F), so we chose 1 U/μL as the optimal concentration.

Finally, the temperature was optimized, as it is a critical parameter in the CRISPR system. Cas12a has been shown to perform optimally within a temperature range of 37°C–42°C (Chen et al., 2018), whereas Cas13a typically exhibits optimal activity around 37°C, although specific temperatures can vary depending on the source strain. For instance, Cas13a derived from bacterial species of the genus Leptotrichia exhibits high activity within the range of 37°C–42°C (Abudayyeh et al., 2017). Therefore, a temperature gradient of 35°C–45°C was established to determine the optimal reaction temperature based on these characteristics. The graph shows that the normalized PL intensity was strongest at both 37°C and 39°C, with no statistically significant difference between the two (Figure 4G), Therefore, 37°C was used in the subsequent development of the one-pot RPA-Cas12a/13a assay.

### 3.5 Sensitivity and specificity assessment

**FIGURE 5:**
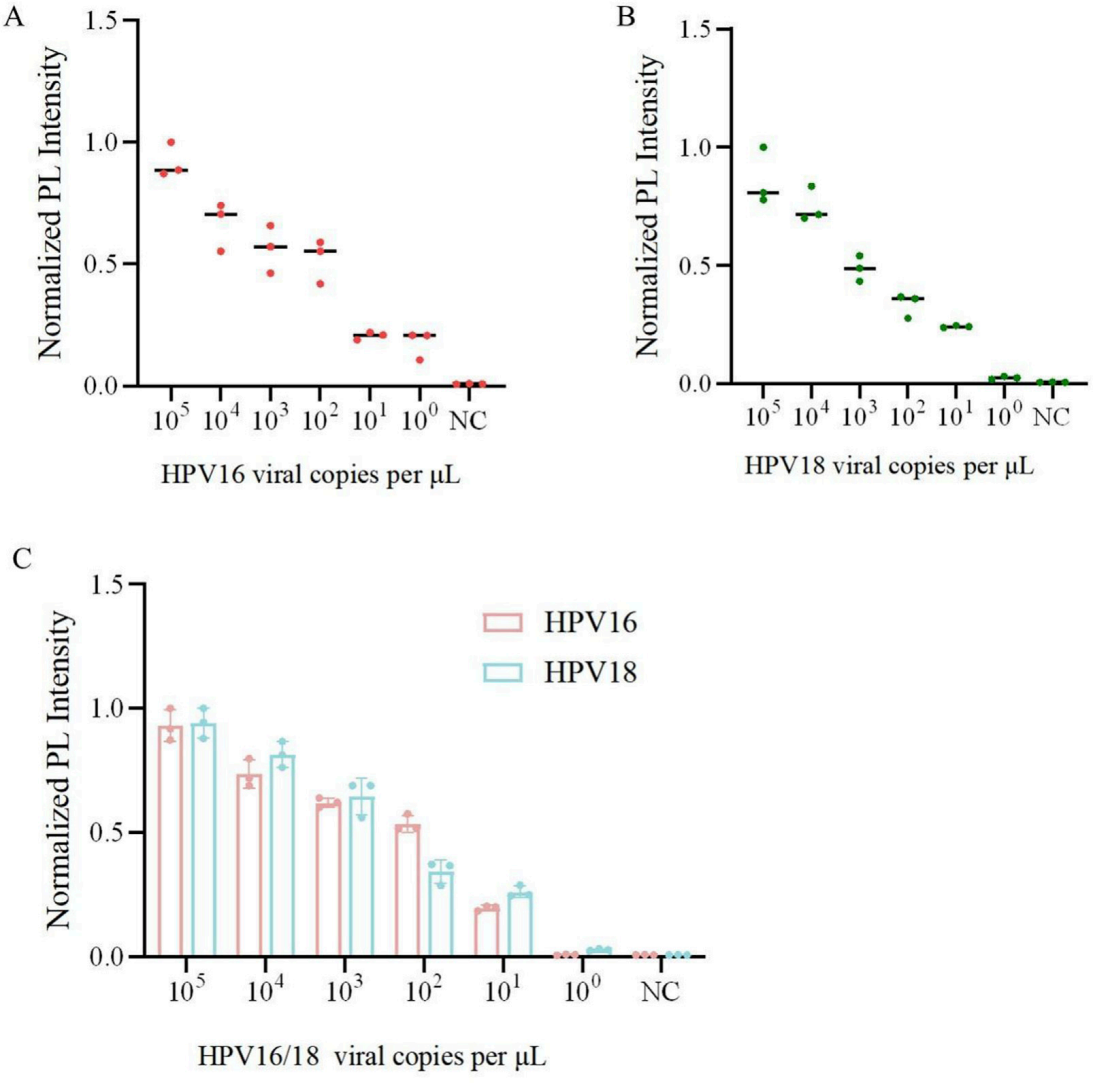
Sensitivity and specificity analysis of one-pot RPA-Cas12a/13a assay. **(A)** Sensitivity of the one-pot Cas12a cutting system for detection of the HPV16. **(B)** Sensitivity of the one-pot Cas13a cutting system for the detection of HPV18. **(C)** Sensitivity of the one-pot RPA-Cas12a/13a dual system for detection of HPV16/18.

**FIGURE 6:**
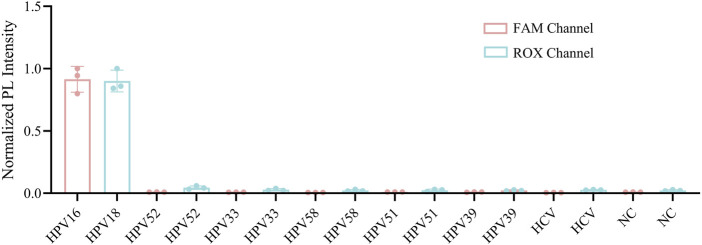
Specificity assessment of the one-pot RPA-Cas12a/13a assay. The specificity was tested with DNA samples from HPV 16, 18, 33, 52, 39, 51, 58 and RNA samples from HCV.

To further validate the sensitivity and specificity of the one-pot Cas12a/13a assay, we performed a gradient dilution of HPV16/18 DNA. The lowest detection limit of the one-tube method for Cas12a to detect HPV16 was 10^0^ copies/μL, while for Cas13a detecting HPV18, it was 10^1^ copies/μL (Figures 5A,B). The results indicated that the one-pot dual-channel system method could detect at least 10^1^ copies/μL of HPV16 and HPV18 (Figure 5C). The sensitivity of our method was significantly improved compared to PCR. To assess the specificity of the one-pot Cas12a/13a method, we selected DNA of other HR-HPV types and RNA of HCV for detection. We found that fluorescent signals were generated exclusively in samples containing the HPV16/18 target genes. No fluorescent signals were detected in samples containing other viruses or types of HPV, indicating that our detection system exhibits excellent specificity and no cross-reactivity (Figure 6). In summary, the method we developed had good sensitivity and specificity.

### 3.6 Examination of clinical sample

**FIGURE 7:**
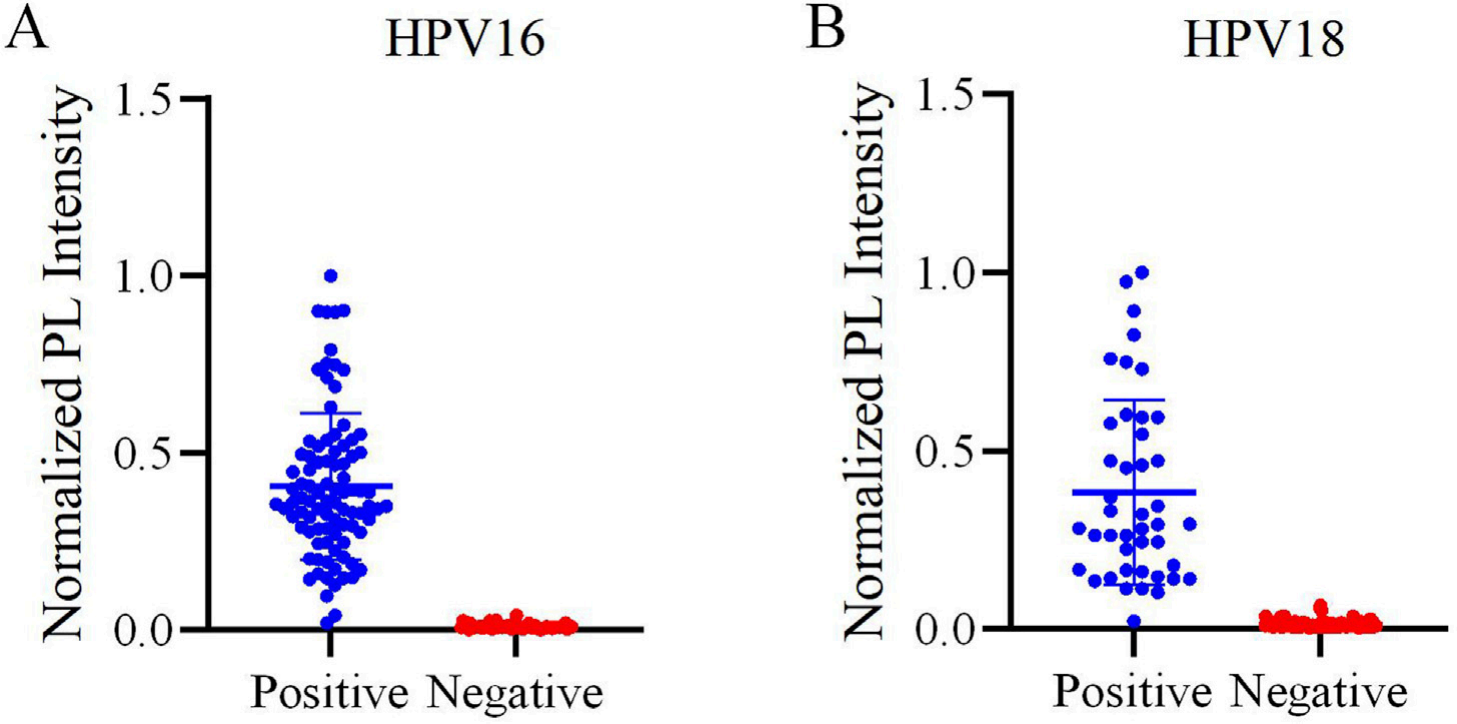
Evaluation of the one-pot RPA-Cas12a/13a assay on HPV samples. **(A)** Scatter plot of the normalized PL intensity of 150 clinical samples tested with the one-pot RPA-Cas12a/13a assay. HPV16-positive samples are indicated by blue dots, and HPV16-negative samples are indicated by red dots. **(B)** Scatter plot of the normalized PL intensity of 150 clinical samples tested with the one-pot RPA-Cas12a/13a assay. HPV18-positive samples are indicated by blue dots, and HPV18-negative samples are indicated by red dots.

This study validated the reliability of the reaction system using 150 clinical vaginal secretion samples. The distribution of HPV16 and HPV18 detections is shown in Figure 6. The results of the scatterplots showed a total of 91 samples positive for HPV16 and 43 samples positive for HPV 18, which included samples that were positive for both HPV 16 and 18 (Figures 7A,B). Most of the samples successfully detected fluorescent signals in the FAM and ROX channels, indicating that our method can effectively identify HPV16 and HPV18. And PCR detected 86 positive samples for HPV16, 37 samples positive for HPV18, 7 positive samples for both HPV16 and HPV18, and 20 negative samples. When compared with PCR results, although our method in three of these samples did not match the PCR results, the overall sensitivity and specificity of our method were 97.69% and 100%, respectively, and the total kappa value was 0.967, indicating that the method demonstrated near agreement with PCR (Table 2).

**TABLE 2:** Comparison of testing methods for 150 clinical samples.

One-pot RPA-Cas12a/13a	qPCR				Total	Sensitivity	Specificity	Kappa value
	HPV16(+)	HPV18(+)	HPV16/18(+)	Negative				
HPV16(+)	84	0	0	0	84	97.67%	100%	0.941
HPV18(+)	0	36	0	0	36	97.30%	100%	0.962
HPV16/18(+)	0	0	7	0	7	100%	100%	1.000
Negative	2	1	0	20	23	—	100%	—
Total	86	37	7	20	150	97.69%	100%	0.967

## 4 Discussion

Cervical cancer is increasingly affecting younger populations, but the development of HR-HPV-associated cervical cancer often remains asymptomatic in the early stages (Cai et al., 2022; Judah et al., 2022). Overlooking HR - HPV may trigger the carcinogenic transformation of the cervix. As a result, there is an urgent requirement for a simple and rapid diagnostic test capable of reliably detecting HR-HPV16/18, which is essential for enabling early intervention and preventing the progression of cervical cancer.

Currently, the combination of isothermal amplification technology and CRISPR system has gained increasing popularity in viral detection due to its convenience and high efficiency. Considerable research has been conducted on HPV typing detection. For instance, Liu et al. developed a multiplex RPA-CRISPR/Cas12a approach for detecting multiple HPV types, yet it was unable to identify specific types (Liu et al., 2024). Other studies have incorporated various amplification techniques and CRISPR/Cas12a/13a systems into HPV detection, utilizing the cleavage properties of these two proteins to specifically detect HPV16/18 (Zheng et al., 2022; Zhou et al., 2023). In contrast to Liu’s study, the latter ones could identify specific types and achieve cleavage in both systems. Nevertheless, all these methods divided the amplification and CRISPR reactions into two steps, which not only heightens the risk of aerosol contamination and complicates the experimental procedure but also increasing manual handling and costs. Thus, we established a one-pot RPA-Cas12a/13a dual-gene assay system to address the need for a simple, rapid and inexpensive solution for HPV16/18 testing.

The entire detection process of the one-pot RPA-Cas12a/13a dual-gene assay system can be completed within 40 min at 37°C. Moreover, the sensitivity of the assay for both genes can reach 10 copies/µL. Furthermore, unlike previously reported dual-channel assay platforms, this method enables real-time monitoring of amplification results in a short period of time, eliminating the need to open the reaction vial at the end of the process. This feature allows for amplification and protein cleavage to occur within a single tube, thus simplifying the workflow and minimizing the risk of aerosol contamination. We further validated a large number of clinical samples to verify the reliability of our methods. Additionally, specificity analysis demonstrated no cross-reactivity with other viruses or different types of HPV, indicating that this platform holds great promise for clinical applications.

Based on the comparison presented in Supplementary Table S1, one-pot CRISPR-based detection systems, including our method, exhibit several distinct advantages over traditional two-step protocols: (1) a simplified workflow with reduced risk of contamination; (2) shorter assay times; (3) improved suitability for point-of-care testing (POCT); (4) comparable or even superior sensitivity. Despite these advantages, challenges remain for POCT implementation. To enhance the robustness of the assay for point-of-care testing POCT applications, these components are physically separated prior to the reaction and are briefly centrifuged to initiate mixing in our current design to avoid the risk of premixing. Most existing dual-channel one-pot assays rely on an intermediate centrifugation step (Wang et al., 2023; Hu et al., 2025), because premature mixing of Cas proteins, crRNA, and target nucleic acids can result in the formation of a trimeric complex, which interfere with the RPA amplification process by binding to and cleaving amplicons, thereby compromising assay performance (Lu et al., 2022). Recent findings suggest that the key difficulty of blocked amplification in CRISPR one-pot reactions was broken by slowing down Cas nuclease cleavage kinetics or optimizing the reaction steps (Tong et al., 2024). This insight offer a promising pathway for the future development of fully integrated one-pot CRISPR diagnostic platforms.

In conclusion, we developed a novel CRISPR/Cas-based assay for the rapid and highly sensitive nucleic acid detection of HPV16/18. Our method provides valuable technical support for early cervical cancer screening and HPV infection detection. Additionally, this approach can be integrated with microfluidics to enable the detection of all high-risk HPV types. By precisely designing reaction zones and partitioning channel, the operational steps could be simplified for POCT. This study, however, does have certain limitations. Compared with the single-channel system, the sensitivity of the one-pot RPA-Cas12a/13a system is somewhat diminished. In the one-pot dual-channel CRISPR assay, the reaction systems compete for resources. The two detection channels share limited resources such as enzymes, substrates, and buffers within the same reaction tube (Ding et al., 2024), and there is also the potential for primer interference (Wang et al., 2021). For DNA extraction from clinical samples, we employed an automated nucleic acid extractor, which is faster but expensive and bulky, thereby restricting its use in point-of-care testing or field applications. Moreover, we only detected HPV16/18, which could not cover all HR-HPV. Future research could focus on developing quantitative assays based on CRISPR systems, and the integration of microfluidic technology could be considered to achieve the typing detection of all HR-HPV.

## Data Availability

The datasets presented in this study can be found in online repositories. The names of the repository/repositories and accession number(s) can be found in the article/Supplementary Material.
