# Supplementary material for: Development of a one-pot RPA-cas12a/13a assay for simultaneous detection of HPV16 and HPV18
Source: Front Bioeng Biotechnol. 2025 Jul 17;13:1608301. doi: 10.3389/fbioe.2025.1608301 (PMC12310682; doi:10.3389/fbioe.2025.1608301)
Supplement: Supplementary file 6 [file DataSheet1.docx]

Supplementary Material

## Supplementary Figures


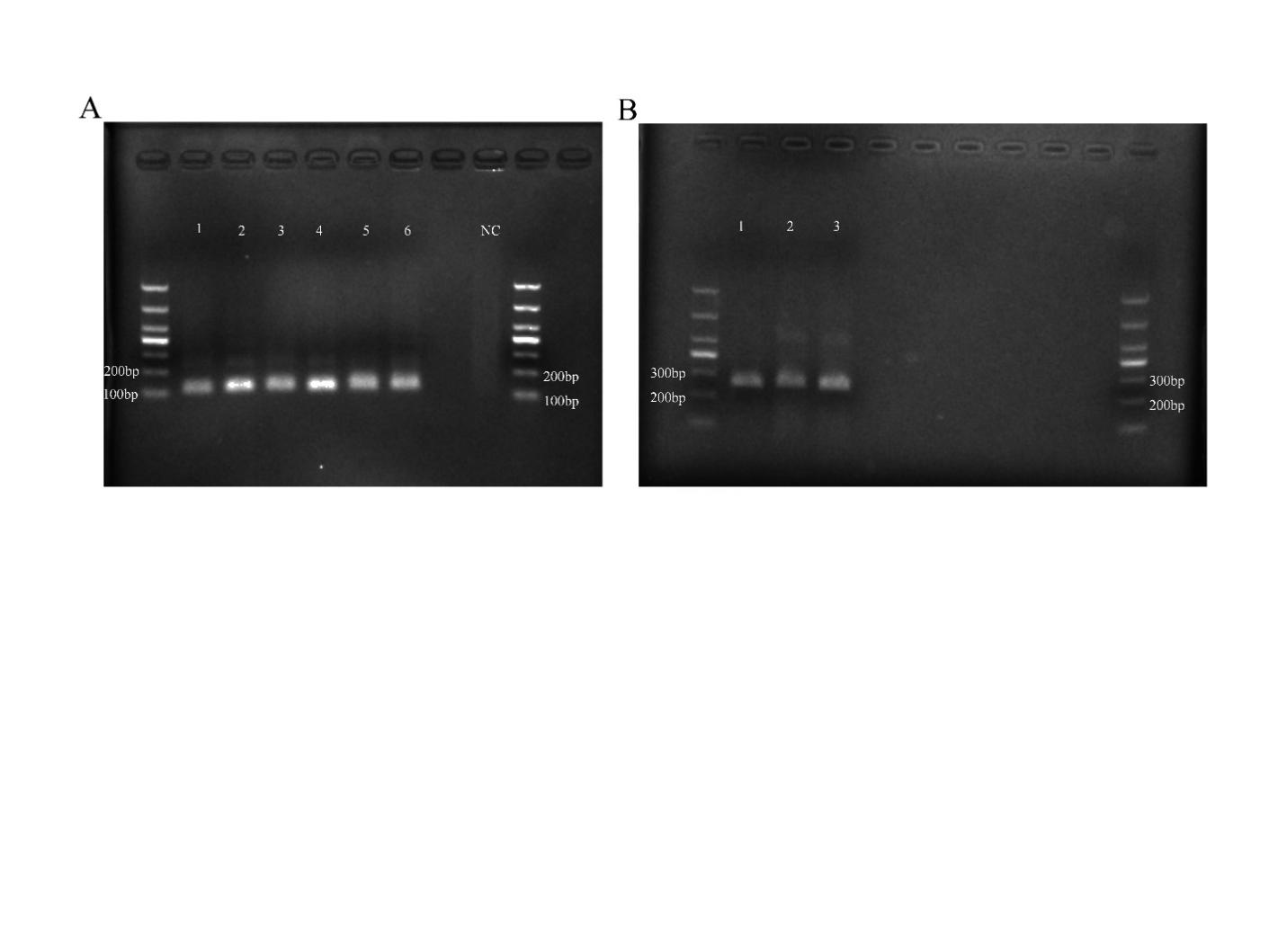


**Supplementary Figure 1.** RPA primer screening. (A) Results of RPA primer amplification gel agarose electrophoresis of HPV18 L1 gene. Lane 1 represents HPV18 primer F1/R1. Lane 2 represents HPV18 primer F2R2. Lane 3 represents HPV18 primer F3/R3. Lane 4 represents HPV18 primer F2/R1. Lane 5 represents HPV18 primer F2R3. Lane 6 represents HPV18 primer F3/R1. NC indicates nuclease-free water. (B) Results of RPA primer amplification gel agarose electrophoresis of HPV16 L1 gene. Lane 1 represents HPV16 primer F1/R1. Lane 2 represents HPV16 primer F2R2. Lane 3 represents HPV16 primer F3/R3.

Supplementary table 1. Systematic comparison of this study with currently reported detection methods.

| Methods | Time | Sensitivity | readout | Tagets | Operation steps | Clinical sample |
| --- | --- | --- | --- | --- | --- | --- |
| Our method | 40min | 10 copies | Fluorescence | HPV16/18 | One-pot | 150 |
| Zheng et al.(Zheng et al., 2022) | 60min | 10^0^ copies | Visual Fluorescence | HPV16/18 | two step | 55 |
| Tian et al.(T et al., 2022) | 90min | 8 copies | Visual Fluorescence | SARS-CoV-2 ASFV | two step | 67 |
| Ding et al.(Ding et al., 2024) | 35min | 20 copies | Fluorescence and LFA | CaMV35S and NOS | One-pot | 24 |
| Cheng et al.(Cheng et al., 2023) | 50min | 1-10 copies | Fluorescence and LFA | SASR-CoV-2 and  MTB | One-pot | 65 |
| Jiang et al. (Jiang et al., 2024) | 50min | 0.24 copies for the FHV-1 and 5.5 copies for the FCV | Fluorescence and LFA | FHV-1 and FCV | two step | 56 |

Reference

Cheng, M., Tan, C., Xiang, B., Lin, W., Cheng, B., Peng, X., et al. (2023). Chain hybridization-based CRISPR-lateral flow assay enables accurate gene visual detection. *Anal Chim Acta* 1270, 341437. doi: 10.1016/j.aca.2023.341437

Ding, L., Wang, X., Chen, X., Xu, X., Wei, W., Yang, L., et al. (2024). Development of a novel Cas13a/Cas12a-mediated “one-pot” dual detection assay for genetically modified crops. *J Adv Res*, S2090-1232(24)00311–4. doi: 10.1016/j.jare.2024.07.027

Jiang, F., Liu, Y., Yang, X., Li, Y., and Huang, J. (2024). Ultrasensitive and visual detection of feline herpesvirus type-1 and feline calicivirus using one-tube dRPA-Cas12a/Cas13a assay. *BMC Vet Res* 20, 106. doi: 10.1186/s12917-024-03953-9

T, T., Z, Q., Y, J., D, Z., and X, Z. (2022). Exploiting the orthogonal CRISPR-Cas12a/Cas13a trans-cleavage for dual-gene virus detection using a handheld device. *Biosensors & bioelectronics* 196. doi: 10.1016/j.bios.2021.113701

Zheng, X., Li, Y., Yuan, M., Shen, Y., Chen, S., and Duan, G. (2022). Rapid detection of HPV16/18 based on a CRISPR-Cas13a/Cas12a dual-channel system. *Anal. Methods* 14, 5065–5075. doi: 10.1039/D2AY01536F
